# Development of an Omnidirectional-Capable Electromagnetic Shock Wave Generator for Lipolysis

**DOI:** 10.1155/2017/9258512

**Published:** 2017-05-24

**Authors:** Ming Hau Chang, San Yih Lin

**Affiliations:** Department of Aeronautics and Astronautics, National Cheng Kung University, No. 1, University Road, Tainan City 701, Taiwan

## Abstract

Traditional methods for adipose tissue removal have progressed from invasive methods such as liposuction to more modern methods of noninvasive lipolysis. This research entails the development and evaluation of an omnidirectional-capable flat-coil electromagnetic shock wave generator (EMSWG) for lipolysis. The developed EMSWG has the advantage of omnidirectional-capable operation. This capability increases the eventual clinical usability by adding three designed supports to the aluminum disk of the EMSWG to allow omnidirectional operation. The focal pressures of the developed EMSWG for different operating voltages were measured, and its corresponding energy intensities were calculated. The developed EMSWG was mounted in a downward orientation for lipolysis and evaluated as proof of concept. In vitro tests on porcine fatty tissues have been carried out. It is found that at a 6 kV operating voltage with 1500 shock wave exposures, a 2 cm thick subcutaneous hypodermis of porcine fatty tissue can be ruptured, resulting in a damaged area of 1.39 mm^2^. At a 6.5 kV operating voltage with 2000 shock wave exposures, the damaged area is increased to about 5.20 mm^2^, which can be enlarged by changing the focal point location, resulting in significant lipolysis for use in clinical applications.

## 1. Introduction

Human obesity is problematic from the viewpoints of both health and aesthetics. Previously, removing excessive fatty tissue involved invasive liposuction methods which posed a risk of excessive bleeding. Side effects such as cellulite might be induced [1–3]. On the other hand, noninvasive methods of lipolysis can lower the risks and are preferred [4–6]. Common noninvasive appliances currently used for body contouring are ultrasonic devices, such as Liposonix® (Solta Medical Inc., USA), UltraShape® (Syneron and Candela Inc., USA) and Cellactor® (Storz Medical Inc., CH) [7–10]. A continuous repetition of high intensity-focused ultrasound might induce lesions or overheating which leads to cavity generation and tissue dehydration [11]. Similarly, low-level laser therapy (LLLT) is a new modality for body contouring. However, some common side effects of LLLT include pain, scarring, and skin pigmentation [12].

The medical application of extracorporeal shock waves for ailments such as renal stones, gallbladder stones, and musculoskeletal disorders has been quite successful [13–17] for the past 20 years or more. Studies on medical applications of shock waves in diverse areas are progressing, such as the treatment of myocardial ischemia [18, 19], diabetes, atherosclerosis, and venous insufficiency as well as in revascularization [20] and soft wound healing [21] and in research on the proliferation of mesenchymal stem cells [22]. When shock waves are applied for lipolysis, temperature increase in the fatty tissue treated with focused shock waves is almost negligible [23–25]. There are three types of extracorporeal shock wave lithotripters: electrohydraulic, electromagnetic, and piezoelectric [21, 23, 24]. The mechanism of lysing adipocytes with extracorporeal shock waves is via cavitation produced by shock waves [6, 23, 24]. Cavitation is the process of gaseous bubble formation in liquid. As negative pressure occurs in water, it induces a cavitation phenomenon which results in expanding bubbles and toroidal collapse near the target surface. These bubbles are formed around the focal region of the focused shock wave. When the bubbles collapse, they emit high-speed water microjets which impact nearby adipocyte membranes [26, 27].

Liang et al. previously had used extracorporeal shock waves to rupture porcine fatty tissues [28, 29]. Their in vitro test results showed that lipolysis by shock waves was feasible. In Liang's works, the electromagnetic shock wave generator (EMSWG) was arranged in a vertical direction so that shock waves were generated and propagated only in an upward direction. For clinical purposes, a unidirectional operation shock wave generator is inconvenient for medical treatment. In their EMSWG, a shock wave is generated by an accelerated moving metal disk in water. The moving disk is returned to the original position by gravity. This limitation is remedied in this work.

In this study, an electromagnetic-type shock wave generator is constructed for lipolysis using a similar mechanism to existing extracorporeal shock wave generator design [6, 13], but having an advantage of possible omnidirectional operation. The efficacy of the refined device is evaluated by measuring the focal pressure of the focused shock wave and by calculating the corresponding energy intensity. Finally, the histological effects on the treated porcine fat tissues are examined.

## 2. Experimental Methods

### 2.1. Experimental Setup

**Figure 1:**
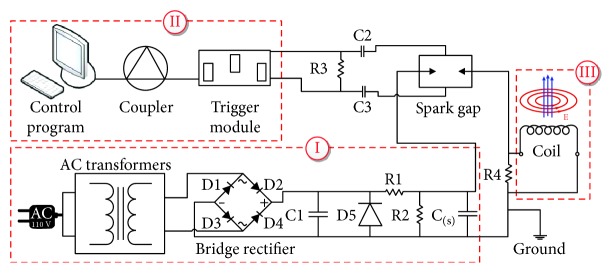
Experimental setup schematic for EMSWG.

The setup of an experimental lipotripter is briefly described below. There are three major parts of the experimental system: (I) a power charging system, (II) a signal control system, and (III) a shock wave generator. The system is depicted in Figure 1.

The power charging system converts alternating current (AC) voltage into direct current (DC) voltage in the range of 0–6.5 kV using an autotransformer and a bridge rectifier. The DC power is stored in several high-voltage capacitors connected to a triggered spark gap (PerkinElmer Inc., USA). The triggered spark gap can be operated in the voltage range of 5–15 kV, which is suitable for capacitor-switching applications such as medical lithotripters.

The signal control system consists of a computer, an analog output module (NI 9263, National Instruments, USA), and a trigger module (PerkinElmer Inc., USA). The signal control program is written using the LabVIEW package (National Instruments, USA). The control program has several settable parameters, such as operating voltage, frequency, and total number of shock wave exposures. The trigger module allows the triggering of the spark gap connected to the shock wave generator.

The electromagnetic shock wave generator in this study consists of a flat coil electromagnet, an aluminum disk, an acoustic lens, and a soft cover. The soft cover comes into contact with the patient. The aluminum disk is accelerated to generate shock waves in liquid when the flat coil is charged. For durability, the acoustic lens is designed to be flat on one side and parabolic on the other to avoid brittleness stemming from the thinness of the lens. The effective focus is found experimentally by locating the peak pressure point of a focused shock wave. The acoustic lens in this study has an effective focal length equal to its geometric focal length of 9 cm [29].

A 30 × 30 × 30 cm rectangular tank was constructed and filled with reverse osmosis (RO) water to accommodate the aforementioned shock wave generator for pressure measurements. RO water is used because it has few impurities to interfere with shock wave transmission. A polyvinylidene difluoride (PVDF) needle-type pressure sensor (SN 1841, Precision Acoustics Inc., UK) was connected through a DC coupler to measure the peak pressure at the focal point. The DC coupler interfaced with an oscilloscope (Agilent Technologies Co., USA) to display the voltage waveform. The measured voltage wave data allowed the calculation of peak pressure and associated energy intensity. The rectangular water tank was reutilized for the lipolysis experiments on the porcine fatty tissues. After hematoxylin and eosin staining, treated tissues were histologically examined to see the effects of the shock waves.

### 2.2. The Electromagnetic Shock Wave Generator

#### 2.2.1. Components of the EMSWG

**Figure 2:**
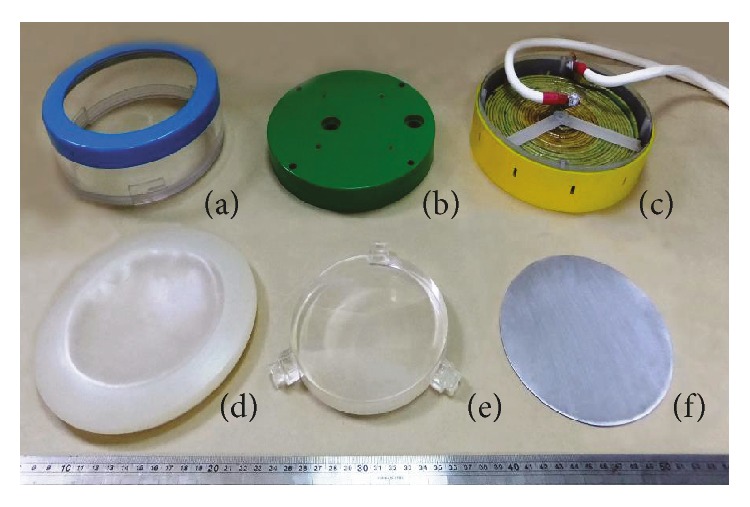
Components of EMSWG: (a) acrylic container, (b) coil cover, (c) coil and coil housing, (d) soft cover, (e) acoustic lens, and (f) aluminum disk.

The functional elements of the EMSWG consist of a circular acrylic container (Figure 2(a)), a flat-shaped electromagnetic coil inside the coil housing (Figure 2(c)), a soft cover (Figure 2(d)), an acoustic lens (Figure 2(e)) and a 1 mm 6061-0 aluminum disk (Figure 2(f)). A coil cover (Figure 2(b)) protects the 22-turn electromagnetic coil. The aluminum disk abuts the coil housing. The acoustic lens with a radius of 6.1 cm is fixed at a distance of approximately 9 mm from the aluminum disk. The circular acrylic container filled with RO water serves as the shock wave medium housing. One end of the container can be screwed onto the coil housing. The other end is connected to the flexible soft cover which physically contacts the target tissue.

#### 2.2.2. Omnidirectional EMSWG

**Figure 3:**
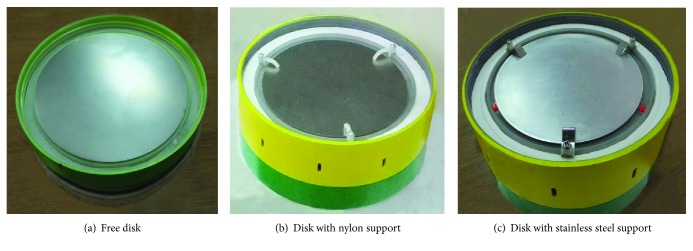
Aluminum disks without and with supports: (a) free disk only having upward arrangement, (b) nylon, and (c) steel supports used for aluminum disk.

For clinical use, omnidirectional operation of an EMSWG would be much more convenient. To this end, the EMSWG is designed to maintain viability of the shock wave generation in various orientations. Though in this work, only the downward orientation is tested to determine initial feasibility of the proposed design. The design uses three small equidistant supports (Figures 3(b) and 3(c)) to constrain the free aluminum disk (Figure 3(a)). Two support materials were tested. The first design incorporated small curved nylon rods as supports, as shown in Figure 3(b). Unfortunately, the nylon rods would deform after about 500 shock waves due to material fatigue. Thus, three 301 stainless steel supports were used in the revised design, as shown in Figure 3(c). These steel supports did not deform after repeated use.

### 2.3. Pressure Measurements

#### 2.3.1. Measurement of Peak Pressures

**Figure 4:**
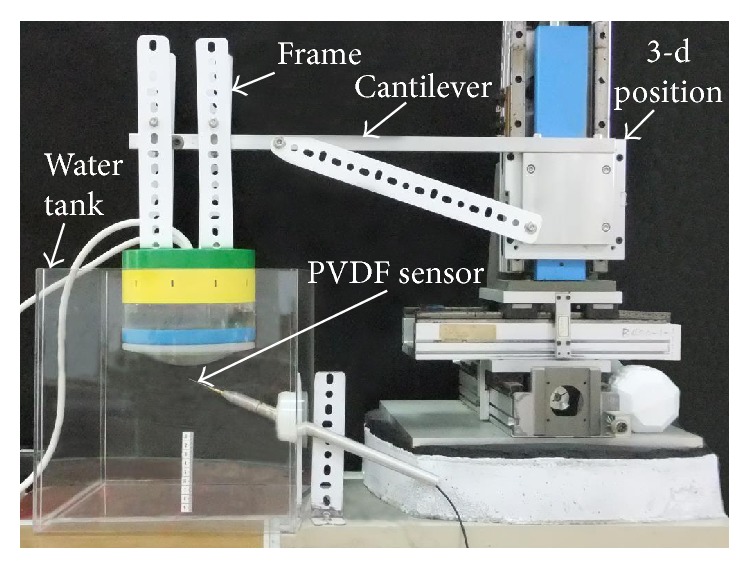
Experimental setup for pressure measurements.

For measuring the peak pressures of focused shock waves, the omnidirectional-capable EMSWG was suspended by a cantilevered three-dimensional positioning platform in a water tank, as shown in Figure 4. The needle tip of a 0.5 mm PVDF hydrophone was placed at the effective focal point of the acoustic lens. The probe sensitivity is about 300 nV/Pa, and the sensitivity tolerance variation is approximately ±3 dB. The operating voltage of the omnidirectional-capable EMSWG was set in the range of 5–6.5 kV with increments of 0.5 kV. Twenty pressure measurement samples were taken for defining the focal pressure at the shock wave exposure frequency of 1 Hz for each tested operating voltage. After obtaining the peak pressure, energy intensity was calculated.

#### 2.3.2. Calculation of Energy Intensity

Energy intensity is defined as the integral of pressure squared and divided by sound impedance.

(1) $$\begin{array}{c} I=\left[\frac{1}{\rho c}\right]\cdot \int p^{2}dt, \end{array}$$

where *I* is the energy intensity, *ρ* the water density, *p* the pressure, *t* the time, and *c* the sound speed in water.

### 2.4. In Vitro Tests on Porcine Fatty Tissues

**Figure 5:**
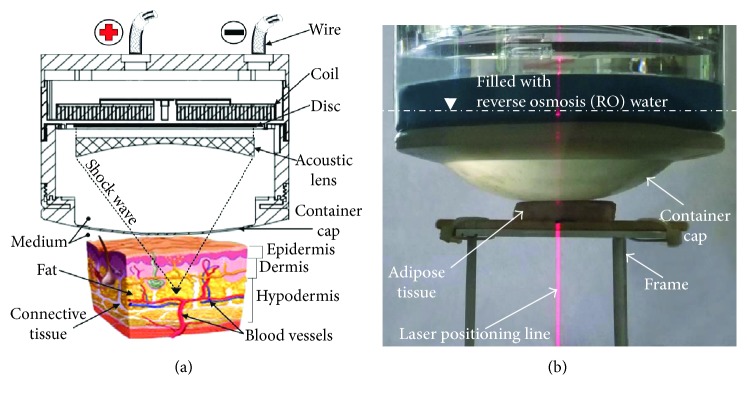
(a) Schematic diagram of an omnidirectional EMSWG and (b) setup used for in vitro experiments.

Since the structure of porcine fatty tissue is similar to that of humans, tissues of the domestic pig (*Sus scrofa domestica*) were used in this study. Normal human skin consists of the epidermis, dermis, and hypodermis, as seen in Figure 5(a). The epidermis and dermis combined have a thickness of 2–4 mm. Human fat cells are mainly stored in the hypodermis, at about 1-2 cm in depth. This corresponds to the treatment range of clinical devices such as the UltraShape® (about 1.5 cm below the skin) and Liposonix (about 1.3 cm below the skin).  Figure 5 shows a cross section diagram of the developed omnidirectional-capable EMSWG. The developed EMSWG is oriented so that the target adipose tissue is beneath the soft cover. The EMSWG and target tissue were submerged into the water tank. A red 650 nm laser positioning device (>5 mW) was used to mark the focal point location in the treated tissue, as shown in Figure 5(b).

### 2.5. Histological Examination

**Figure 6:**
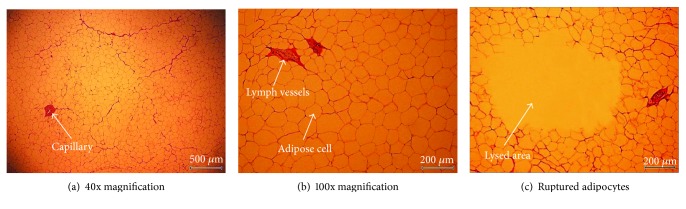
Histological examination after H&E staining of normal porcine adipocyte specimen at (a) 40x magnification. Examination of (b) normal and (c) shock wave-treated specimens at 100x magnification.

In order to examine the damage extent of the treated porcine fatty tissues, the 2 cm treated tissues were fixed and dehydrated by being packed in a cassette and submerged in a formalin solution for three days. After the processes of clearing and infiltration, the treated tissues were embedded and sliced into 3 *μ*m thick slices. The final process of hematoxylin and eosin was carried out for histological examination through a microscope.  Figure 6 shows the comparison of the normal and ruptured porcine adipocytes.  Figure 6(a) shows a ~3.5 × 2.5 mm^2^ picture of normal porcine adipocytes at 40x magnification.  Figure 6(b) shows a ~1.4 × 0.99 mm^2^ picture of normal porcine adipocytes at 100x magnification. In Figure 6(b), the regular arrangement of the normal adipocytes is clearly seen. In Figure 6(c), the empty region indicates ruptured adipocytes.

## 3. Results and Discussion

### 3.1. Pressure Measurements

**Figure 7:**
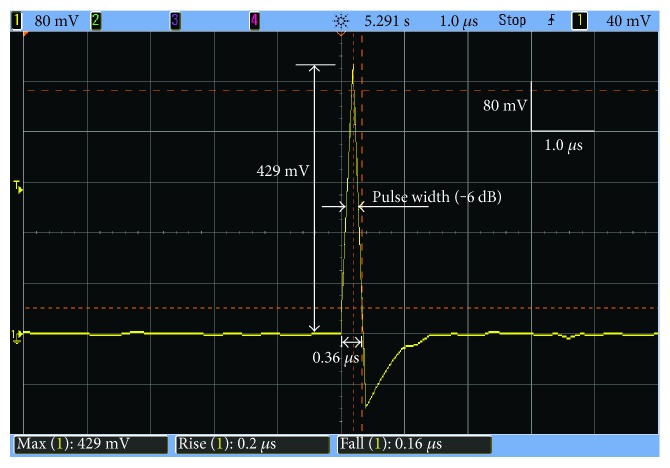
Focal pressure characteristics of a single pressure pulse measured by a PVDF hydrophone.

The developed EMSWG is arranged in a downward position. The measured focal pressure profile of a typical focused shock wave is shown in Figure 7. The 429 mV peak of the pressure pulse corresponds to the peak pressure of 1.41 MPa and the energy intensity of 0.081 mJ/mm^2^ at the operating voltage of 6.5 kV. The duration time of the focal pressure profile is 0.36 *μ*s. The rise time is 0.2 *μ*s and 0.16 *μ*s for the fall time.

### 3.2. Experimental Results of In Vitro Tests

#### 3.2.1. Effect of Adipose Tissue Thickness

For studying the effect of adipose tissue thickness, the operating voltages were set to 5.5–6.5 kV with increments of 0.5 kV. The RO water medium has an acoustic impedance of approximately 1.4 M rayls, which is close to that of the blood (at approximately 1.6 M rayls). Shock wave experiments without targets served as the control cases. The measured peak pressures for the controls are used for comparison with the adipose tissue experiments. In order to calculate the extent of energy intensity loss through the treated fatty tissue, skinless adipose tissue specimens of three different thicknesses (1, 2, and 3 cm) were selected so that the effective focal point was located at 1-2 cm below the surface of the treated tissue.

**Figure 8:**
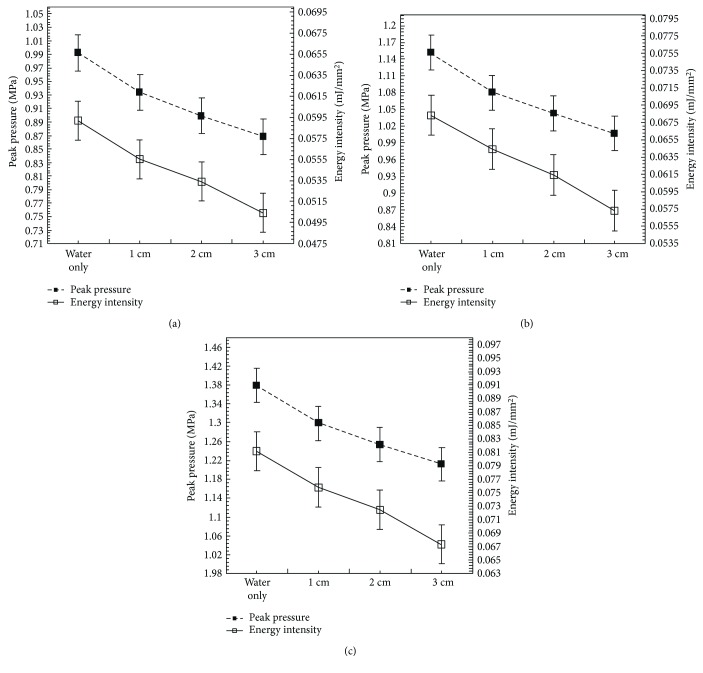
The peak pressure and energy intensity with error bar at the focus for different tested samples of porcine adipose tissues without the skin at operating voltages of (a) 5.5 kV, (b) 6 kV, and (c) 6.5 kV.

The measured peak pressure and energy intensity with error bars at the focus for different tested samples and operating voltages are shown in Figure 8. Note that the PVDF needle hydrophone tip was positioned right below the adipose tissue.  Table 1 shows the measured peak pressures for various treated porcine adipose tissues. Twenty pressure data points were used. The average peak pressure at 5.5 kV for the water case is 0.99 MPa, 0.93 MPa for 1 cm thick adipose tissue, 0.9 MPa for 2 cm thickness, and 0.87 MPa for 3 cm thickness. The recorded peak pressure loss of the 1 cm thick adipose tissue at 5.5 kV is 6.1% less compared to the control, with a 6.1% reduction at 6 kV and 6.5% reduction at 6.5 kV. For 2 cm thick adipose tissue, the measured peak pressure is 0.9 MPa at 5.5 kV, which is a 9.1% reduction compared to the peak pressure of the control case. Operating voltage of 6 kV results in a 9.6% reduction and 6.5 kV results in a 9.4% reduction.

For adipose tissue of 3 cm thickness, the measured peak pressure is 0.87 MPa at 5.5 kV which is a 12.1% reduction compared to the peak pressure of the control case, with a 12.2% reduction at 6 kV, and a 12.3% reduction at 6.5 kV. The corresponding energy intensities for various cases are shown in Table 2. The trends of the peak pressure and energy intensity losses due to adipose tissue thickness are shown in Figure 8.

In the same way, one can study the effect of energy intensity through adipose tissue thickness. It is found that the reduction in the energy intensity for the 1 cm thickness is 5.1% at 5.5 kV, 5.9% at 6 kV, and 6.2% at 6.5 kV. For the 2 cm thickness, the reduction in the energy intensity is 10.2% at 5.5 kV, 10.3% at 6 kV, and 11.1% at 6.5 kV. For the 3 cm thickness, the reduction in the energy intensity is 15.3% at 5.5 kV, 16.2% at 6 kV and 17.3% at 6.5 kV.

For the effects of shock wave exposures over the range of 5.5–6.5 kV, the peak pressure and energy intensity have a proportional relationship regardless of the operating voltage. Both the peak pressure and energy intensity are inversely related versus the specimen thickness. Thus, the effect is proportionally decreased with the increase of tissue thickness. Moreover, it was also found that when using fatty tissues of 3 cm thickness, the energy intensity was reduced by about 10–18%, therefore reducing the performance of lipolysis treatment. In general, human adipose tissues are mainly stored in the hypodermis, so the effective focal point is generally 1-2 cm below the skin of a patient undergoing a clinical application. Therefore, porcine fatty tissues of 2 cm thickness were selected for further study.

#### 3.2.2. Evaluation of the Affected Area

Among noninvasive clinical treatments currently used for lipolysis or body contouring, the main treatment depth for adipose tissue is approximately 2 cm due to the fact that the subcutaneous hypodermis region of the abdomen is located approximately 2 cm deep. For this reason, the specimen size of porcine fatty tissue was set at 2 cm thick. The amount of affected adipose tissue is basically dependent on two parameters: the operating voltage and the number of shock wave exposures.

The adipose tissues treated with shock waves were processed through paraffin block sectioning as well as hematoxylin and eosin staining. The extent of adipose tissue lysis was observed through a microscope. As seen from Figure 6(c), there is a region of ruptured adipose tissue after shock wave treatment. In order to easily identify the region affected by shock waves, the region of ruptured adipose tissue is shaded in subsequent images.

**Figure 9:**
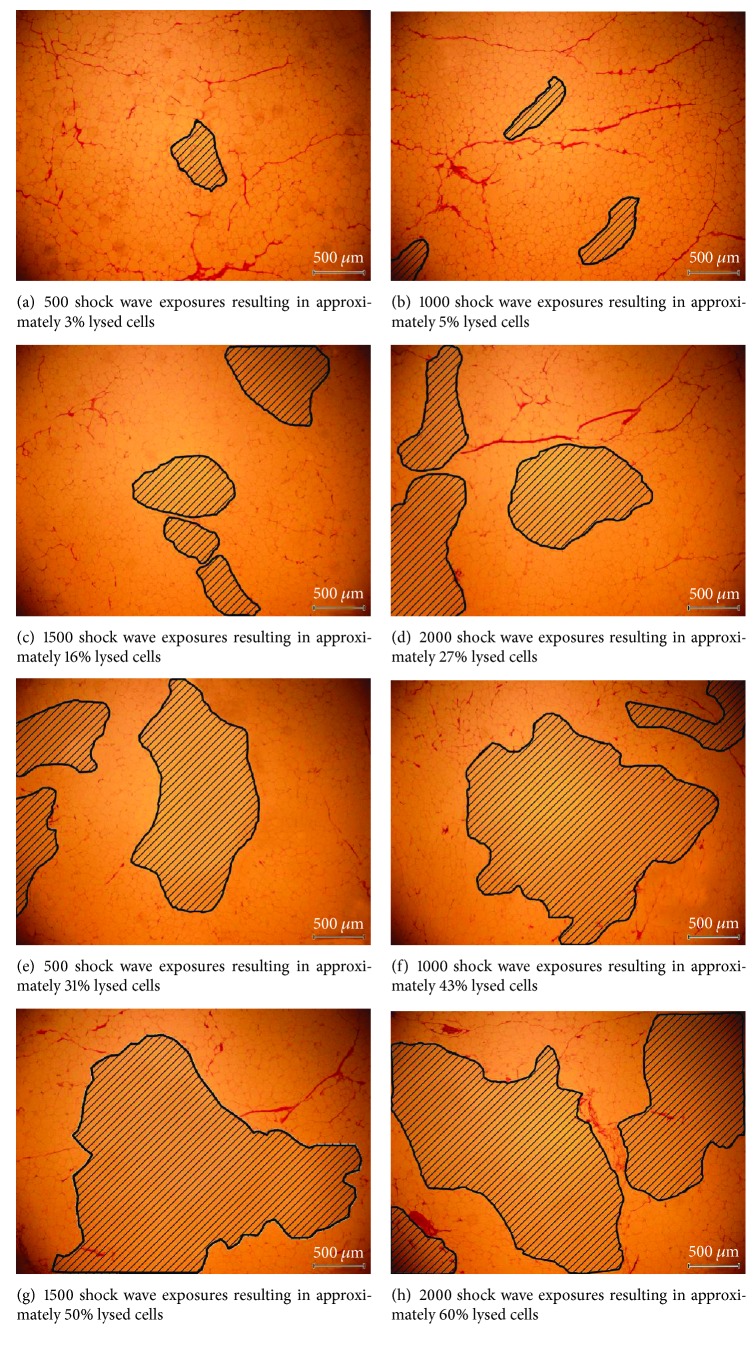
Adipose tissue specimens measuring 3.5 × 2.5 mm^2^ with ruptured adipocytes (shaded areas) after H&E staining for various number of shock wave exposures at 6 kV (a–d) and 6.5 kV (e–h). These are representative samples at 2 cm depth with 40x magnification.

In order to estimate the extent of the damaged area, specimens of the treated adipose tissue were examined after H&E staining.  Figure 9 shows images of the ruptured adipose tissue at 40x magnification for different shock wave exposures at 6 kV. The examined adipose tissue specimens were about 3.5 × 2.5 mm^2^ through a microscope. It is found that the lysed (shaded) area is about 0.24 mm^2^, with approximately 28 ruptured adipocytes for 500 shock waves (Figure 9(a)). For 1000 shock waves, the damaged area is at least 0.46 mm^2^, because some damage is out of the image range, with approximately 54 ruptured adipocytes (Figure 9(b)). For 1500 shock waves, the lysed area is at least 1.39 mm^2^, with approximately 164 ruptured adipocytes (Figure 9(c)). For 2000 shock waves, the lysed area totaled at least 2.35 mm^2^, with approximately 270 ruptured adipocytes (Figure 9(d)), as shown in Table 3.

Figures 9(e), 9(f), 9(g), and 9(h) show 40x magnification pictures of the lysed adipose tissues at the 2 cm depth after H&E staining for different shock wave exposures at 6.5 kV. For 500 shock waves, the lysed area is at least 2.73 mm^2^, with approximately 320 ruptured adipocytes (Figure 9(e)). For 1000 shock waves, the lysed area is at least 3.80 mm^2^, with approximately 448 ruptured adipocytes (Figure 9(f)). For 1500 shock waves, the lysed area is at least 4.36 mm^2^, with approximately 512 ruptured adipocytes (Figure 9(g)). Finally, for 2000 shock waves, there is at least 5.20 mm^2^ lysed area, with approximately 610 ruptured adipocytes, as shown in Figure 9(h). Table 3 summarizes the data pertaining to Figure 9.

## 4. Conclusion

In consideration of the clinical use of an electromagnetic shock wave generator for lipolysis, an omnidirectional EMSWG proof of concept has been developed and evaluated. The developed EMSWG can be operated not only in an upward position but also in a downward position. Hence, the functionality of the EMSWG is enhanced.

The performance of the omnidirectional-capable EMSWG has been histologically examined through in vitro testing of the lipolysis effect on porcine adipose tissues located at a depth of 2 cm. The affected region of the treated adipose tissue is dependent on the operating voltage and the number of shock wave exposures. Effective lipolysis results at operating voltages of at least 6 kV. If the operating voltage is less than 6 kV, adipose tissue damage is almost negligible. At operating voltages of 6 kV or greater, some extent of damage on the treated adipose tissues is observable. In general, the use of operating voltages higher than 6.5 kV may cause side effects on the patient skin. However, additional shock wave exposure at a lower operating voltage can achieve the same treatment outcome. For 2 cm thick fatty tissues at 6 kV operating voltage, 500, 1000, 1500, and 2000 shock wave exposures yield lysed areas of percentages of about 2.7%, 5.3%, 15.9%, and 26.9%, respectively. As expected, increasing the operating voltage enhances lipolysis effects. At 6.5 kV, 500, 1000, 1500, and 2000 shock wave exposures yield larger lysed area of percentages of about 31.2%, 43.4%, 49.8%, and 59.4%, respectively.

These initial results are promising, and this approach warrants further study for possible clinical application as a noninvasive omnidirectional electromagnetic-type shock wave generator for lipolysis.

## Figures and Tables

**Table 1:** Average peak pressures of focused shock waves for various specimens.

Specimen	RO water				1 cm thick adipose tissue			2 cm thick adipose tissue			3 cm thick adipose tissue		
Operating voltage (kV**)**	5	5.5	6	6.5	5.5	6	6.5	5.5	6	6.5	5.5	6	6.5
Average (MPa) ± SD	0.87 ± 0.004	0.99 ± 0.003	1.15 ± 0.067	1.38 ± 0.06	0.93 ± 2.37	1.08 ± 2.57	1.29 ± 1.74	0.9 ± 1.1	1.04 ± 2.77	1.25 ± 0.91	0.87 ± 1.07	1.01 ± 1.19	1.21 ± 1.07
Energy intensity change (%)	n/a				−6.1	−6.1	−6.5	−9.1	−9.6	−9.4	−12.1	−12.3	−12.3

**Table 2:** Average energy intensities of focused shock waves for various specimens.

Specimen	RO water				1 cm thick adipose tissue			2 cm thick adipose tissue			3 cm thick adipose tissue		
Operating voltage (kV)	5	5.5	6	6.5	5.5	6	6.5	5.5	6	6.5	5.5	6	6.5
Average (mJ/mm^2^) ± SD	0.052 ± 0.6*e*−3	0.059 ± 0.4*e*−3	0.068 ± 0.5*e*−3	0.081 ± 0.5*e*−3	0.056 ± 0.28*e*−3	0.064 ± 0.27*e*−3	0.076 ± 0.18*e*−3	0.053 ± 0.19*e*−3	0.061 ± 0.15*e*−3	0.072 ± 0.21*e*−3	0.05 ± 0.29*e*−3	0.057 ± 0.20*e*−3	0.067 ± 0.24*e*−3
Energy intensity change (%)	n/a				−5.1	−5.9	−6.2	−10.2	−10.3	−11.1	−15.3	−16.2	−17.3

**Table 3:** Effect of lipolysis at 6 kV and 6.5 kV with various number of shock wave exposures on 3.5 × 2.5 mm^2^ sized specimens.

Operating voltage (kV)	6				6.5			
Number of shock wave exposures	500	1000	1500	2000	500	1000	1500	2000
Lysed area (mm^2^)	0.24	0.46	1.39	2.35	2.73	3.80	4.36	5.20
Lysed area (%)	2.7	5.3	15.9	26.9	31.2	43.4	49.8	59.4
Ruptured adipocytes	28	54	164	277	321	448	512	611
